# Supplementary material for: Naturalistic drug cue reactivity in heroin use disorder: orbitofrontal synchronization as a marker of craving and recovery
Source: medRxiv. 2024 Apr 19:2023.11.02.23297937. Originally published 2023 Nov 3. Preprint. [Version 2] doi: 10.1101/2023.11.02.23297937 (PMC10635268; doi:10.1101/2023.11.02.23297937)
Supplement: 1 [file NIHPP2023.11.02.23297937V2-supplement-1.pdf]

medRxiv preprint doi: https://doi.org/10.1101/2023.11.02.23297937; this version posted April 19, 2024. The copyright holder for this preprint (which was not certified by peer review) is the author/funder, who has granted medRxiv a license to display the preprint in perpetuity.
It is made available under a CC-BY 4.0 International license .

## Supplement

### Exclusion criteria

Exclusion criteria for all participants were the following: 1) DSM-5 diagnosis for schizophrenia or neurodevelopmental disorder; 2) Head trauma with loss of consciousness longer than 30 min; 3) History of central nervous system disease; 4) Cardiovascular, metabolic, endocrinological, oncological, autoimmune, and infectious diseases including Hepatitis B and C or HIV/AIDS; 5) Metal implants or other MR contraindications (including pregnancy); 6) Court mandated treatment. We did not exclude for DSM-5 diagnosis of a drug use disorder other than opiates as long as heroin was the primary drug of choice/reason for treatment-seeking since iHUD commonly use alcohol, amphetamines, benzodiazepines, other sedatives, cocaine, and marijuana in addition to heroin. Exclusion criteria for the HC were the same, except history of any drug use disorder was prohibitive.

### Diagnostic details in individuals with heroin use disorder

Comorbidities in iHUD included major depressive disorder (n=6), cocaine use disorder (n=5), post-traumatic stress disorder (n=2), sedative use disorder (n=3), cannabis use disorder (n=2), alcohol use disorder (n=3), generalized anxiety disorder (n=1), meth/amphetamine use disorder (n=1), and panic disorder (n=1).

### Controlling for group differences in sample characteristics

The HUD and HC groups differed significantly in years of education, verbal IQ, Beck's Depression Inventory, Beck's Anxiety Inventory, Fagerström Test for Nicotine Dependence, the Short Michigan Alcohol Screening Test and years of regular marijuana use (Table 1). To inspect their putative contribution to results, we tested these variables' correlations with the root-mean-square signal at significant time points (see Identifying synchronized TR's and region-specific reactivity) in the ROIs that showed higher drug bias in HUD at baseline (yielding 392 tests: 28 ROIs x 7 control variables x 2 groups). None of these ROIs showed a significant correlation when correcting for multiple comparisons (with FDR correction). Similarly, we tested correlations between each of these demographic variables and ISC scores in the OFC and did not find significant correlations (Table S6). Overall, these control analyses suggest that our main results (the drug bias measured here and the OFC as a marker of craving) were not driven by group differences in the above variables.

### Treatment-specific details

Study participants were randomized into Mindfulness Oriented Recovery Enhancement and support group therapy sessions, both received in addition to medication-assisted inpatient treatment. The former group sessions involved mindfulness-based self-awareness and emotion regulation training (e.g., cognitive reappraisal of negative and savoring of positive thoughts/contexts). These strategies were delivered with the goal of diminishing drug cue-reactivity while enhancing natural reward processing and cognitive control over craving and drug-seeking behaviors (see[73] for therapy details). The support group included structured, therapist-guided and addiction-related psychoeducation, emotional expression, and discussions. In both groups, subjects were instructed to supplement the group sessions with daily 15-min independent practice sessions guided by audio instructions or journaling, respectively.

### Reverse correlation with control movie labels

To test for generic effects of salient movie labels on reverse correlation results, we generated two sets of control labels for the movie, one for high/low brightness and one for high/low loudness (see methods for labeling details). Repeating the whole-brain reverse correlation analyses with these labels revealed group differences between HUD and HC, but none overlapped with the ROIs identified by the drug bias analysis. This result suggests that the drug bias effects cannot be explained by labeling any salient feature of the movie.

medRxiv preprint doi: https://doi.org/10.1101/2023.11.02.23297937; this version posted April 19, 2024. The copyright holder for this preprint
(which was not certified by peer review) is the author/funder, who has granted medRxiv a license to display the preprint in perpetuity.
It is made available under a CC-BY 4.0 International license .

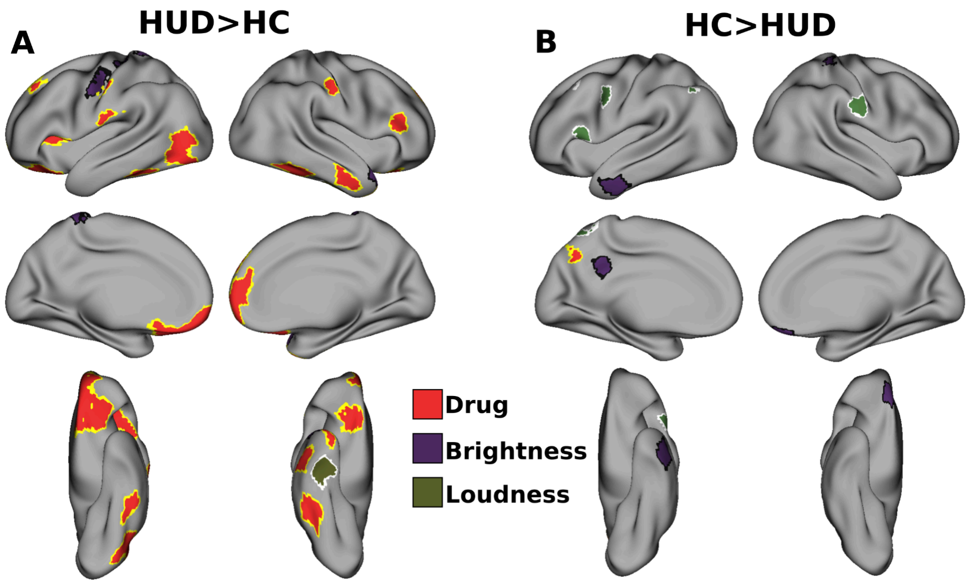


Supplemental Figure 1. Reverse correlation maps with loudness and brightness labels do not overlap with drug labels. ROIs showing significantly higher bias in HUD (A) or HC (B) towards drug (red), high brightness (purple), or high loudness (green) content at the baseline session. Note that significant ROIs do not overlap between labels, particularly in the OFC.

**Testing changes in control measures between sessions**

Our primary behavioral outcome of interest was craving. We had three craving measures collected both before and after treatment: scene-induced craving, pre-movie craving, and movie-induced craving; a fourth measure, the Heroin Craving Questionnaire, was only collected at baseline. Given that we only expect meaningful craving signals in HUD (no HC participants reported drug craving in any of these measures), we performed one-sample paired tests (follow-up minus baseline) in HUD for each of these craving measures; the resulting p-values were corrected for multiple comparisons with FDR correction. Only scene-induced craving showed a significant reduction after treatment (Table S1). Although the movie was drug-related, this result suggests that fine-grained scene-level craving, which more closely assesses cue-reactive dynamic fluctuations in craving, are needed to demonstrate changes in craving with treatment; these same craving measures were also the only to correlate with the OFC (main text). To test the specificity of craving in iHUD as a behavioral marker of treatment outcome, we also ran one-sample paired tests on five other control measures that were collected at both sessions and found no other significant changes with treatment in HUD (Table S2 ) or in HC (Table S3, S4).

| Craving variable | test | statistic | p | mean | std | q |
|---|---|---|---|---|---|---|
| Scene-induced craving | wilcoxon | 34 | 0.008 | -1.5 | 2.8 | 0.024 |
| Pre-movie craving | ttest | -2 | 0.052 | -0.74 | 1.9 | 0.072 |
| Movie-induced craving | wilcoxon | 29 | 0.072 | -0.56 | 1.5 | 0.072 |

Table S1. Statistical tests for between session craving effects in HUD. Paired (follow-up minus baseline) tests were performed against the null hypothesis of zero mean. q indicates FDR-corrected p-values.

| Control variable | test | statistic | p | mean | std | q |
|---|---|---|---|---|---|---|
| Scene-induced emotional increase | ttest | -0.92 | 0.37 | -0.63 | 3.7 | 0.62 |
| Scene-induced emotional decrease | ttest | -0.22 | 0.83 | -0.13 | 3.2 | 0.83 |
| Movie item recognition | ttest | -1.5 | 0.14 | -0.033 | 0.12 | 0.35 |
| Beck's Depression Inventory | ttest | -1.8 | 0.09 | -2.8 | 8.3 | 0.35 |
| Beck's Anxiety Inventory | ttest | 0.48 | 0.63 | 0.69 | 7.6 | 0.79 |
| Methadone dose | ttest | 0.0 | 1.0 | 0.0 | 26.0 | 1.0 |

Table S2. Statistical tests for between session effects in control variables in HUD. Paired (follow-up - baseline) tests were performed against the null hypothesis of zero mean. q indicates FDR-corrected p-values.

medRxiv preprint doi: https://doi.org/10.1101/2023.11.02.23297937; this version posted April 19, 2024. The copyright holder for this preprint (which was not certified by peer review) is the author/funder, who has granted medRxiv a license to display the preprint in perpetuity.
It is made available under a CC-BY 4.0 International license .

| Craving variable | test | statistic | p | mean | std | q |
|---|---|---|---|---|---|---|
| Scene-induced craving | wilcoxon | 2.5 | 0.79 | -0.042 | 1.2 | 0.79 |
| Pre-movie craving | wilcoxon | 0 | 0.32 | -0.21 | 1 | 0.48 |
| Movie-induced craving | ttest | 1.7 | 0.1 | 0.83 | 2.4 | 0.3 |

Table S3. Statistical tests for between session craving effects in HC. Paired (follow-up - baseline) tests were performed against the null hypothesis of zero mean. q indicates FDR-corrected p-values.

| Control variable | test | statistic | p | mean | std | q |
|---|---|---|---|---|---|---|
| Scene-induced emotional increase | ttest | -1.6 | 0.12 | -1.4 | 4 | 0.6 |
| Scene-induced emotional decrease | wilcoxon | 15 | 0.37 | 0.5 | 2.4 | 0.64 |
| Movie item recognition | ttest | 0.8 | 0.43 | 0.015 | 0.091 | 0.64 |
| Beck's Depression Inventory | ttest | 0.48 | 0.64 | 0.28 | 2.9 | 0.64 |
| Beck's Anxiety Inventory | ttest | 0.56 | 0.58 | 0.32 | 2.8 | 0.64 |

Table S4. Statistical tests for between session effects in control variables in HC. Paired (follow-up - baseline) tests were performed against the null hypothesis of zero mean. q indicates FDR-corrected p-values.

**Correlation between OFC signal and control behavioral variables**

We hypothesized that OFC ISC scores would specifically correlate with measures of craving. We had four different craving measures at baseline (scene-induced craving, pre-movie craving, movie-induced craving, and the Heroin Craving Questionnaire). Of these craving measures, scene-induced and pre-movie craving were intercorrelated at baseline (r:0.68, p:0.000045) as well as in their delta between sessions (r:0.6, p:0.00093). We tested for correlations between all four of these craving measures and OFC ISC scores at baseline and for correlations between the session deltas of each of these craving measures (except the Heroin Craving Questionnaire that was only collected at baseline) and the session deltas of the OFC ISC scores. FDR correction was applied over the seven correlations tested. Only the correlation between the session deltas with scene-induced craving survived multiple comparisons correction ($q<0.05$); at baseline, scene-induced craving showed a trend toward significance ($p<0.05$) (Table S5).

To test the specificity of the relationship between OFC ISC scores and craving, we also ran correlations between the OFC ISC scores and several control measures. Here we included three measures from the same post-movie survey used for the scene-induced craving measure (ratings for whether a subject was able to increase or decrease their emotional reaction to a scene, item recognition memory scores), demographic or psychometric variables that differed between the groups (Beck's Depression Inventory, Beck's Anxiety Inventory, Fagerström Test for Nicotine Dependence, Short Michigan Alcohol Screening Test, years of regular marijuana use, verbal IQ, years of education), and measures other than craving that specifically related to heroin use (years of regular heroin use, Heroin Severity of Dependence Scale, Short Opiate Withdrawal Scale, days of abstinence from heroin, days of heroin use in the past 30 days). We also tested correlations between the session deltas of OFC ISC scores and the control variables that were collected at both sessions. FDR correction was applied over all control correlations tested. None of these control variables survived FDR correction ($q<0.05$), although the session delta for Beck's Depression Inventory showed a trend towards significance ($p<0.05$).

| Craving variable | Session | r | p | q |
|---|---|---|---|---|
| Scene-induced craving | Baseline | 0.38 | 0.04 | 0.14 |
| Pre-movie craving | Baseline | 0.29 | 0.12 | 0.27 |
| Movie-induced craving (post-pre) | Baseline | -0.15 | 0.44 | 0.61 |
| Heroin Craving Questionnaire | Baseline | 0.079 | 0.68 | 0.68 |
| Scene-induced craving | Delta (follow-up - baseline) | 0.54 | 0.0023 | 0.016 |
| Pre-movie craving | Delta (follow-up - baseline) | 0.12 | 0.54 | 0.63 |
| Movie-induced craving (post-pre) | Delta (follow-up - baseline) | -0.27 | 0.16 | 0.27 |

Table S5. Correlations between OFC ISC and craving measures in HUD. For rows involving deltas between sessions, the difference between follow-up and baseline was used for both the craving measures and OFC ISC scores. r indicates Pearson correlation coefficient, q indicates FDR-corrected p values.

| Control variable | Session | r | p | q |
|---|---|---|---|---|
| Scene-induced emotional increase | Baseline | 0.15 | 0.44 | 0.93 |
| Scene-induced emotional decrease | Baseline | -0.034 | 0.86 | 0.93 |
| Movie item recognition | Baseline | 0.028 | 0.88 | 0.93 |
| Beck's Depression Inventory | Baseline | 0.28 | 0.14 | 0.93 |
| Beck's Anxiety Inventory | Baseline | 0.046 | 0.81 | 0.93 |

medRxiv preprint doi: https://doi.org/10.1101/2023.11.02.23297937; this version posted April 19, 2024. The copyright holder for this preprint (which was not certified by peer review) is the author/funder, who has granted medRxiv a license to display the preprint in perpetuity.
It is made available under a CC-BY 4.0 International license .

| | | | | |
|---|---|---|---|---|
| Fagerström Test for Nicotine Dependence | Baseline | -0.036 | 0.85 | 0.93 |
| Short Michigan Alcohol Screening Test | Baseline | -0.0096 | 0.96 | 0.96 |
| Years of regular marijuana use | Baseline | -0.22 | 0.26 | 0.93 |
| Verbal IQ | Baseline | 0.035 | 0.86 | 0.93 |
| Years of Education | Baseline | 0.22 | 0.26 | 0.93 |
| Years of regular heroin use | Baseline | -0.34 | 0.073 | 0.73 |
| Heroin Severity of Dependence Scale | Baseline | -0.13 | 0.5 | 0.93 |
| Short Opiate Withdrawal Scale | Baseline | 0.039 | 0.84 | 0.93 |
| Days of abstinence from heroin | Baseline | 0.054 | 0.78 | 0.93 |
| Days of heroin use in past 30 days | Baseline | 0.048 | 0.81 | 0.93 |
| Methadone Dose | Baseline | 0.33 | 0.13 | 0.73 |
| Scene-induced emotional increase | Delta (follow-up-baseline) | -0.12 | 0.54 | 0.93 |
| Scene-induced emotional decrease | Delta (follow-up-baseline) | -0.17 | 0.37 | 0.93 |
| Movie item recognition | Delta (follow-up-baseline) | -0.12 | 0.51 | 0.93 |
| Beck's Depression Inventory | Delta (follow-up-baseline) | 0.43 | 0.021 | 0.42 |
| Beck's Anxiety Inventory | Delta (follow-up-baseline) | 0.061 | 0.75 | 0.93 |
| Methadone dose | Delta (follow-up-baseline) | 0.43 | 0.087 | 0.62 |

Table S6. Correlations between OFC ISC and control measures in HUD. For rows involving deltas between sessions, the difference between follow-up and baseline was used for both the craving measures and OFC ISC scores. r indicates Pearson correlation coefficient, q indicates FDR-corrected p values.
